# Inhibition of Demineralization of Dentin by Fluoride-Containing Hydrogel Desensitizers: An In Vitro Study

**DOI:** 10.3390/jfb13040246

**Published:** 2022-11-16

**Authors:** Yasuhiro Matsuda, Bayarchimeg Altankhishig, Katsushi Okuyama, Hiroko Yamamoto, Katsuaki Naito, Mikako Hayashi, Hidehiko Sano, Sharanbir K. Sidhu, Takashi Saito

**Affiliations:** 1 Division of Clinical Cariology and Endodontology, Department of Oral Rehabilitation, School of Dentistry, Health Sciences University of Hokkaido, Tobetsu 061-0293, Japan; 2 Department of Dental Materials Science, Asahi University School of Dentistry, Mizuho 501-0296, Japan; 3 Department of Restorative Dentistry and Endodontology, Osaka University Graduate School of Dentistry, Suita 501-0296, Japan; 4 Department of Restorative Dentistry, Hokkaido University Graduate School of Dental Medicine, Sapporo 060-8586, Japan; 5 Oral Bioengineering, Institute of Dentistry, Queen Mary University of London, London E1 2AD, UK

**Keywords:** hypersensitivity, hydrogel, root caries, fluoride, PIXE/PIGE

## Abstract

Several desensitizers routinely used clinically for dentin hypersensitivity are expected to inhibit demineralization. This study aimed to evaluate the effectiveness of sealing materials in inhibiting demineralization and increasing fluorine (F) uptake by acid-treated root surfaces. Five noncarious extracted human teeth were used to produce specimens. Three different fluoride-containing materials, namely “MS Coat F” (MS), “MS Coat Hys Block Gel” (HS), and CTX2 Varnish (FV), were used herein. Each material was applied to the demineralized root surface. Single sections were obtained from each specimen. All surfaces of each specimen, except the polished surface, were covered with wax and immersed in an automatic pH cycling system for 2 weeks. Fluorine and calcium distributions in the carious lesions of each specimen were evaluated using proton-induced gamma emission (PIGE) and X-ray (PIXE) techniques, respectively. Dentin demineralization was analyzed using transverse microradiography (TMR) before and after pH cycling. µPIXE/PIGE analysis demonstrated that all sample groups showed increased fluoride uptake on the root surface. TMR analysis revealed that both HS and FV showed significantly lower integrated mineral loss values than the control group. All three samples demonstrated a tendency towards increased fluoride uptake from fluoride-containing hypersensitivity desensitizers and a demineralization inhibition effect on root dentin.

## 1. Introduction

Untreated caries is an important disease for human health management [1,2]. The percentage of people over 80 years of age with 20 or more permanent teeth is increasing, and maintaining over 20 teeth is associated with healthy aging [3]. The number of remaining teeth in the elderly is expected to increase, and the preservation of these teeth is important for the overall health of society. One of the characteristics of the remaining teeth in the elderly is the exposure of the root surfaces due to gingival recession, which increases the risk of root surface caries and dentin hypersensitivity.

Furthermore, the proportion of hydroxyapatite in dentin and cementum is lower than that in enamel. Owing to the lower acid resistance of dentin and cementum, demineralization at the root surface tends to progress approximately twice as fast as that in enamel [4]. Therefore, it is essential to take proactive preventive measures against root caries, with the aim of suppressing the progression of caries as much as possible, and to implement appropriate restorative measures where necessary. 

Various control materials have been widely applied to treat dentin hypersensitivity. External stimuli, from various sources such as plaque, can trigger dentin hypersensitivity due to exposed dentin tubules. The rapid movement of tissue fluid in the dentin tubules generates hydrodynamic pressure, which mechanically deforms nerve endings in the dentin tubules and causes pain. The following methods are used to treat dentin hypersensitivity: (1) raising the threshold of the pulpal nerve to blunt it; (2) coagulating proteins in the tubules to keep the intra-tubular fluid from moving; and (3) sealing the dentin tubules. Potassium ions are well known to depolarize nerves and interfere with nerve transmission. Thus, potassium nitrate is widely used as an agent to treat dentin hypersensitivity. Most potassium nitrate-containing hypersensitivity suppressants are in the form of pastes and are mainly used for daily brushing. Dentin hypersensitivity suppressants with glutaraldehyde, which have a protein coagulation effect, are used only in professional care as they irritate the gingival tissues. 

The most widely used materials in clinical practice are those that seal dentin tubules. Among the materials that seal dentin tubules and suppress sensitivity, some seal dentin tubules with minerals, such as calcium phosphate and fluoro-aluminosilicate glass, while others seal dentin tubules and suppress sensitivity by forming a polymer film in the dentin tubules [5]. Many of these materials, such as fluoride varnishes, contain fluoride. Fluoride (F) is expected to improve the acid resistance of enamel and dentin and to promote remineralization [6,7,8,9,10,11]. It is widely used for caries prevention and its effectiveness is well established. Fluoride-containing hypersensitivity treatment materials can be expected to not only seal dentin tubules but also improve the acid resistance of dentin and promote remineralization by providing a source of F. 

It would be beneficial in clinical practice if hypersensitivity-control materials could control both root surface hypersensitivity and caries. In particular, it is desirable to promote the demineralization and remineralization of early caries. Many hypersensitivity-control materials that seal dentin tubules seal them quickly by the application of the material. However, the amount that can be applied to the tooth surface is small because it is usually in a liquid form, and it is challenging to keep it in contact with the tooth surface for an extended period. Recently, gel-type hypersensitivity control materials that contain similar components have been developed. Owing to their gel-like nature, more material can be applied to the tooth surface than in the liquid form. Consequently, gel-type hypersensitivity control materials can act for longer periods and are expected to have a more substantial inhibitory effect on demineralization. Most studies on fluoride-containing hypersensitivity control materials have analyzed the tubule-sealing effect of the applied materials. However, no study has examined F uptake by the applied materials or the demineralization inhibitory effect of the uptake of F. 

We have previously investigated the effects of fluoride-containing hypersensitivity control materials on dentin demineralization. To compare the demineralization and remineralization of dentin, we developed an automatic pH cycle device [12] and examined the demineralization and remineralization of dentin [13,14]. In addition, as reported previously, we mapped the distribution of F in dentin by in-air micro-particle-induced X-ray/gamma-ray emission (PIXE/PIGE) analysis and quantitatively analyzed the amount of fluoride uptake in dentin [6,7,15,16]. 

This study aimed to evaluate the effectiveness of sealing materials in inhibiting demineralization and increasing fluorine uptake by the acid-treated root surfaces by using fluoride-containing materials in different forms. In the gelatinous dentin hypersensitivity-coating material, we investigated the fluoride uptake from hypersensitivity suppressants in demineralized dentin and the effect of demineralization suppression using an in-air µ-PIXE/PIGE analyzer combined with an automated pH cycling environment and transverse microradiography (TMR). 

## 2. Materials and Methods

### 2.1. Specimen Preparation

**Figure 1:**
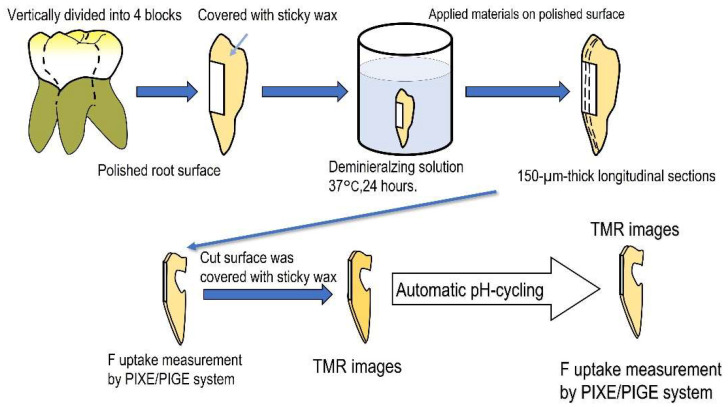
Experimental procedure for fluoride uptake and anti-demineralization analysis.

The experimental procedure is outlined in Figure 1.

Five noncarious extracted human third molars were used in this study. Each tooth was longitudinally divided into a four-specimen block, and the outer root surface was polished using #1000 silicon carbide paper [17]. Specimens were covered with sticky wax on the non-polished surfaces and demineralized in a buffer solution (0.2 M lactic acid, 3.0 mM CaCl_2_, 1.8 mM KH_2_PO_4_, 2% carboxymethyl cellulose, pH 4.5) at 37 °C for 24 h. Three fluoride-containing sealing materials (MS Coat F [MS]), fluoride-containing hydrogel material MSCoatHysBlockGel (Gel Desensitizer) [HS] or fluoride varnish (CTX2 fluoride varnish” [FV]) were used (Table 1). One of these three materials was applied to the polished surface in each specimen-block of the same tooth; the fourth untreated surface served as the control specimen. The specimens were then stored in a phosphate-buffered saline (PBS) solution (pH 7.5) at 37 °C for 24 h. From each block, 2 longitudinal sections (150 µm thick) were prepared. Fluorine and calcium distributions in the carious lesions of each specimen were evaluated using in-air µ-PIXE/PIGE techniques. The cut surfaces of the specimens were covered with sticky wax. Specimens were immersed in an automatic pH cycling system that simulated daily acid changes (pH 4.5–6.8) occurring in the oral cavity for 2 weeks. Caries formation was analyzed using transverse microradiography (TMR). 

This study was approved by the Research Ethics Committee of the Health Science University of the Hokkaido School of Dental Medicine.

### 2.2. Automatic pH Cycling System

**Figure 2:**
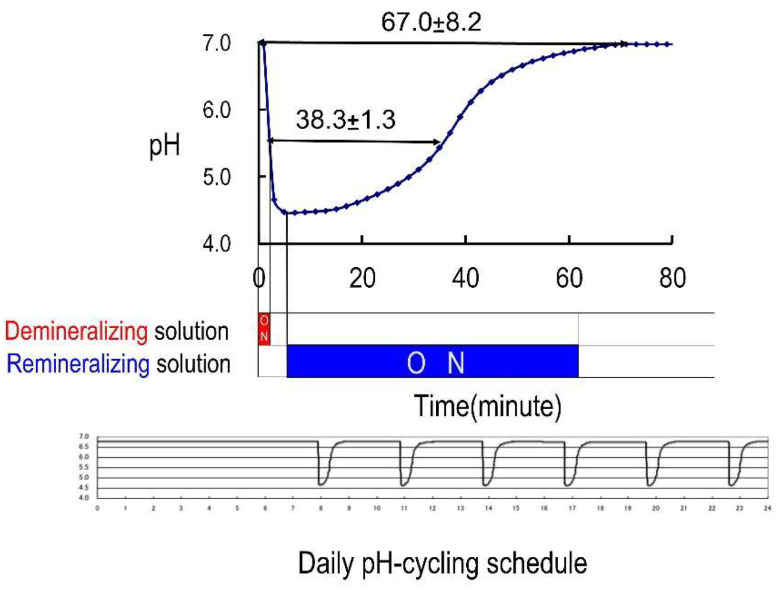
Automatic pH cycling system which provides a severe daily pH-cycle simulation.

Materials applied to the specimen surface were challenged by a simulated daily acid attack occurring on the exposed surface by using an automatic pH cycling system [12]. To simulate daily acid attack in the oral cavity, pH cycling (pH 4.5–6.8) was performed for two weeks by immersing the specimens in an automatic pH cycling system (Figure 2) [6]. The demineralizing solution (pH 4.5) contained 0.2 M lactic acid, 3.0 mM calcium chloride (CaCl_2_), and 1.8 mM potassium dihydrogen phosphate (KH_2_PO_4_); the remineralizing solution (pH 6.8) comprised 0.02 M HEPES, 3.0 mM CaCl_2_, and 1.8 mM KH_2_PO_4_. 

### 2.3. Mineral Loss Analysis

To estimate dentin demineralization, transversal microradiography (TMR) images were obtained using a soft X-ray system (CSM-2, Softex Corporation, Kanagawa, Japan) with an aluminum step wedge and high-resolution photo plates (HRP-SN-2, Konica Minolta, Inc., Tokyo, Japan) before and after four weeks of pH cycling. Transmission electron microscopy (TEM) grids (EM fine grid (F-100), Nissin EM, Tokyo, Japan) were halved and fixed to the control sides of the specimens for superimposition on images obtained using TMR. The exposure was performed for 30 min at 10 kV and 3 mA with a focus-specimen distance of 44 mm. A TEM grid was used to superimpose TMR images. On the TMR images, the average density of the areas with a width of 50 µm was measured at 0.67 µm increments. The integrated mineral loss (ΔIML) was calculated from the mineral profiles obtained before and after pH cycling according to previously reported procedures [18,19,20].

### 2.4. Fluoride Uptake Analyses by PIGE and PIXE Techniques

The wax coatings on the specimen surfaces were removed using xylene. The fluorine and calcium distributions in three specimens from each fluoride-containing material, which were subjected to pH cycling, were evaluated using an air Micro-PIGE/PIXE system at Takasaki Ion Accelerators for Advanced Radiation Application (TIARA) [14,21]. The analytical techniques used were the same as those reported previously [11]. A 3.0 MeV proton beam was extracted from an ion microbeam apparatus. The specimen was attached directly to the window at the end of the microbeam system [21]. The beam spot size was approximately 1 µm in diameter, with a beam current of approximately 100 pA. The maximum scanned area was 1000 µm × 1000 µm. A nuclear reaction (19F (p, ac)160) was used to measure the F concentration, and the gamma rays of this reaction were detected using an 81 cm^2^ NaI detector placed 4 mm behind the specimen. The calcium concentration was measured using proton-induced X-ray emission, which was simultaneously detected with a Si (Li) detector placed in vacuo [21]. The distance and angle of the detector were 118 mm from the target and 40° from the beam axis. For quantitative analysis, the beam intensity was monitored using the X-ray yield from a copper foil by switching the beam onto the foil every 3 s for 30 s.

### 2.5. Estimation of Fluorine Uptake from Materials

F and Ca concentrations were measured in three different 270 µm wide and 270 µm deep areas of each specimen. The measured data were opened using the analysis application [13,14] that converts the data into 128 × 128 pixels images [21]. Fluorine uptake from the sealing material to the dentin surface was also analyzed. The outermost surface of the polished surface was located at a spot containing 5% calcium in the intact dentin. To compare the fluorine uptake, the average fluorine concentration in each specimen was calculated for an area approximately 10 µm from the defined surface. The cumulative amount of F in the outer 100 µm of the lesion was obtained. Two-way ANOVA and Tukey tests were used for analysis (*p* < 0.05).

## 3. Results

### 3.1. Analysis of Fluorine Uptake by the In-Air µPIXE/PIGE Method

**Figure 3:**
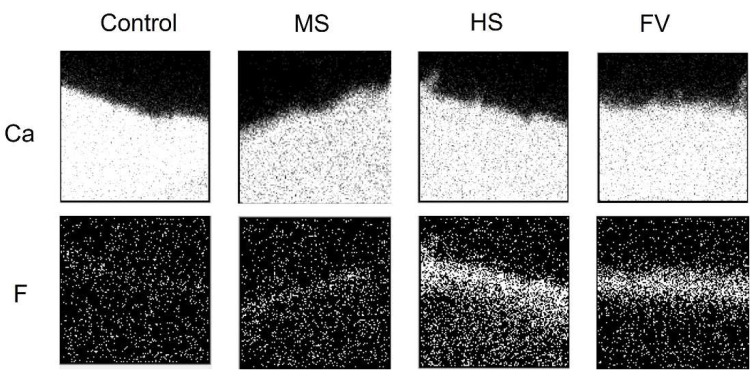
Elemental PIXE map (calcium) and PIGE map (fluorine) of the dentin before the pH-cycle simulation. White dots in the maps (270 µm × 270 µm area) represent PIXE or PIGE signals from calcium, and fluorine, respectively.

**Figure 4:**
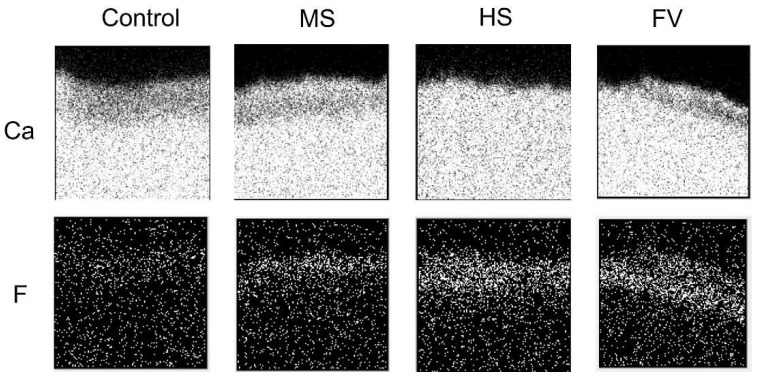
Elemental PIXE map (calcium) and PIGE map (fluorine) of the dentin after the pH-cycle simulation. White dots in the maps (270 µm × 270 µm area) represent PIXE or PIGE signals from calcium, and fluorine, respectively.

In-air µPIXE/PIGE analysis images of cross sections 24 h after the application of each material are shown in Figure 3, and the image after the pH-cycle simulation is shown in Figure 4. 

The areas containing calcium and fluorine are shown in white and the areas not containing calcium and fluorine are shown in black. The calcium distribution image shows the tooth structural area, and the upper surface of the image is the coating surface. Fluoride distribution was not observed in the control group. Fluoride was present in all material groups, and when compared to the calcium distribution image, fluoride was incorporated into dentin. 

In terms of calcium distribution, the control group showed a lower calcified surface layer after the pH cycle compared to that before the pH cycle. The MS group showed a slightly lower calcified image, while the FV group showed a sub-surface decalcified image with a highly calcified layer including fluorine on the surface and a lower calcified layer underneath it. The HS group showed no hypocalcified surface layer.

Fluorine was not detected in the control group before and after pH cycling; in the MS group, a small amount of fluorine was detected in the superficial layer before pH cycling, and a slightly fluorine-permeated image was observed after pH cycling. In the FV and HS groups, fluorine was strongly detected in the superficial layer before pH cycling, and a diffuse image in the deeper areas was observed after pH cycling. FV groups showed a stronger signal than the HS group in the deeper areas.

**Figure 5:**
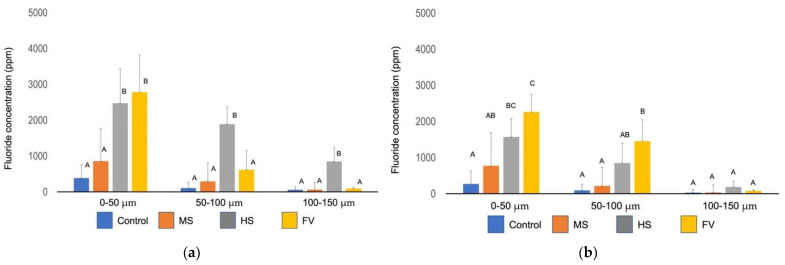
The average amount of fluorine incorporated into the dentin before the pH-cycle (**a**) and after the pH-cycle (**b**). Different letters indicate significant differences (two-way ANOVA and the Tukey tests, *p* < 0.05).

Figure 5a shows the average amount of fluoride at every 50 µm interval from the tooth surface to the interior before the pH cycling. Although there was no significant difference (*p* > 0.05) between the control and MS groups in the 0–50 µm region, the HS and FV groups showed more fluoride uptake than the control group. Only the HS group showed significant fluoride in the 50–100 µm and 100–150 µm intervals from the surface. 

Figure 5b shows the average amount of fluoride at every 50 µm interval from the tooth surface to the interior after pH cycling. The groups with applied material showed greater fluoride uptake than the control group at 0–50 µm from the surface. At 50–100 µm from the surface, the FV group showed more fluoride uptake than the MS and HS groups. There were no significant differences between the material groups and the control group at 100–150 µm from the surface (*p* > 0.05).

### 3.2. Examination of the Amount of Demineralization

**Figure 6:**
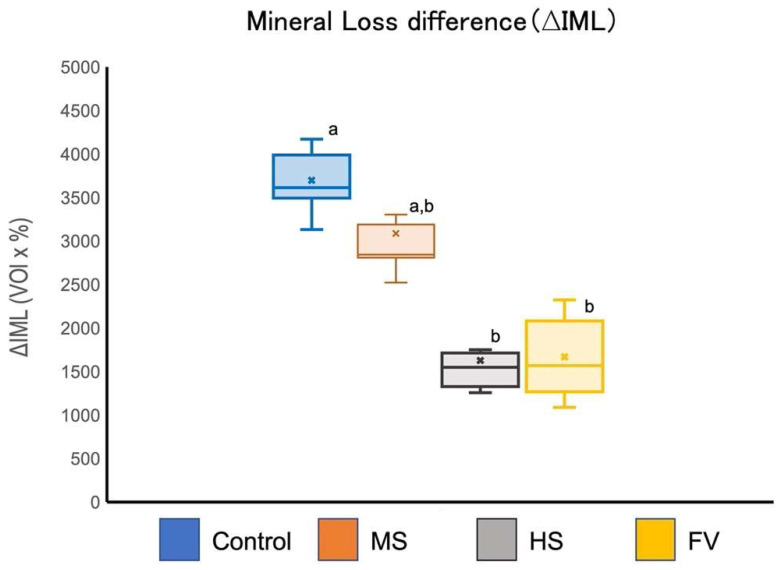
Mineral loss increase (ΔIML) after 2 weeks of cariogenic pH cycling. Different letters indicate significant differences (two-way ANOVA and the Tukey’s tests, *p* < 0.05).

The increase in mineral loss (∆IML) after automatic pH cycling is shown in Figure 6. The ΔIML for groups HS and FV was significantly lower than that for the control group. There was no significant difference in the ΔIML between the MS and control groups. 

## 4. Discussion

The results of this study show that fluoride varnishes and gel-type hypersensitivity suppressants were effective in reducing early root surface caries in demineralized dentin, and that all the materials demonstrated a tendency to promote incorporation of fluoride. Gel-type hypersensitivity suppressants provide fluorine to deeper dentin before pH cycling, and the fluoride varnish provides fluorine with a similar volume of gel-type hypersensitivity suppressants after pH cycling. In addition, the fluoride varnishes and gel-type hypersensitivity suppressants were shown to supply fluorine into the dentin and inhibit dentin demineralization. Fluoride uptake into the superficial dentin layer was observed in all the specimen groups. Quantitative analysis showed that the distribution of fluorine differs depending on the material, especially at depths greater than 100 µm; more fluorine was incorporated into the HS group than into the groups treated with the other materials. In terms of fluorine uptake after pH cycling, the FV group had a significantly higher uptake than the other groups. This result suggests that the fluoride varnish remained on the dentin surface and supplied fluoride, most likely because the varnish is water-repellent and is not easily washed away by water or fluid. Fluoride was found to be more profoundly incorporated in the HS group than in the control group, suggesting that it was flushed out of the dentin by pH cycling. It is considered that MS deposits fluorine-containing structures on the surface layer, and that these thin films repel water and supply fluorine. Furthermore, it has been reported that these structures are not hydrophilic [5]. Both MS and HS materials include a polymer that could potentially generate this thin layer on the dentin. A comparison of mean fluoride concentrations before pH cycling simulation at 50 µm intervals showed a significant difference only in the HS group in the 50–100 µm and 100–150 µm intervals. Conversely, the FV group showed the highest fluorine uptake in the 0–50 µm and 50–100 µm regions. However, in the HS group, fluorine in the dentin was reduced from that before pH cycling but some was retained. A gelatinous dentin hypersensitivity-coating material is a hydrophilic gel that allows a larger amount of material to be retained on the tooth surface compared to other materials. Mitchell et al. reported that the firmer the consistency of the base material in the hypersensitivity control material, the better the sealing property of the dentin tubules and the longer the material could remain in the dentin tubules [22]. The firmer consistency of the base material enabled longer retention in the dentin tubules [22]. In the present study, the HS group with a higher material consistency showed greater acid resistance than the MS and FV groups. It is believed that the demineralization treatment created voids in the dentin, which then facilitated the diffusion of fluorine ions. Demineralized dentin is more susceptible to caries but is also more likely to be penetrated by other minerals.

ΔIML of demineralization and remineralization showed that demineralization was suppressed in the material groups compared with that in the control group. In particular, the HS and FV groups showed a highly calcified superficial layer with fluorine, which may be due to the residual material on the surface inhibiting acid contact; the sodium fluoride that remained with the material also inhibited demineralization. Considering the results of fluoride uptake, it is thought that the demineralization inhibitory effect was caused by the uptake of fluoride into dentin.

Fluoride varnish, which is widely used in clinical practice in developed countries [11,23,24,25,26], is made of highly viscous and hydrophobic pine needles. The hydrophobic film on the varnish is thought to remain in contact on the dentin surface, sealing the dentin tubules and preventing acid-induced demineralization. Generally, fluoride varnish is removed from the tooth surface by brushing after approximately one month [6]. We have previously reported that fluoride varnishes partially remain on the dentin surface after removal of the varnish by water-resistant abrasive paper and simulated brushing and that a demineralization-inhibiting effect was observed on the dentin surface [6]. Fluoride varnish has been reported to contain a high concentration of fluoride, but this fluoride is not eluted in a hydrophilic environment [27]. Therefore, it is thought that it is difficult to supply fluoride to the deep dentin in such a short period (24 h), as in the present study. The gel dentin hypersensitivity treatment material, HS, is in a gel form; thus, it is difficult to dry unlike MS. It is theorized that fluoride may have diffused into the deep dentin because it can remain in contact with the tooth surface in a gel state. Uptake of fluoride deep into the dentin with polymer-based structures, as in MS, is thought to be due to the pH cycle loading that may have promoted remineralization by the fluoride staying deep in the dentin.

Although the fluoride concentrations in the MS, FV, and HS groups were 3000 ppm, 22,600 ppm, and 900 ppm, respectively, the HS group showed the same demineralization inhibitory effects as those in the FV group, which may be due to the difference in the amount of fluoride applied to the tooth surface. Despite fluoride varnish containing a high concentration of sodium fluoride, the amount of fluoride applied to the tooth surface is much smaller than that of the gel dentin hypersensitivity treatment because it is applied thinly to the tooth surface. On the other hand, although the gel dentin hypersensitivity coating material has a low concentration of fluoride because it is in a gel form, it is possible to apply more material to the tooth surface and allow it to stay in place compared to other materials. Comparing the amount of fluoride applied to the MS, FV, and HS groups, the amount applied to the HS group was almost 100 times greater than that in the FV group.

In the prevention of dentin caries, which is primarily a root surface caries lesion, it is important not only to improve acid resistance but also to inhibit the degradation of organic matter such as collagen. It has been reported that the activities of the matrix metalloproteinase (MMP) family of collagen-degrading enzymes and cathepsin, a cysteine protease, are associated with dentin degradation [28]. These proteolytic enzymes are thought to play important roles in dentin caries. The activity of these MMPs is concentration-dependent, is reversibly inhibited by fluoride at 200 ppm or higher, and irreversibly inhibited at 5000 ppm or higher [29]. Zinc and copper also inhibit MMPs [30]. Therefore, the application of fluoride can be expected not only to inhibit demineralization but also to inhibit the degradation of collagen fibers in dentin.

The clinical results of noninvasive treatment of early root surfaces using other topically applied fluoride materials, including fluoride varnish, have also been reported in the literature. In clinical practice, the application of high concentrations of fluoride (5% NaF varnish, 8% SnF_2_ solution) in the early stages of root surface caries has been shown to improve the ratio of soft lesions to hard lesions of the root surface caries [31]. The application of low-concentration (250 ppm fluoride solution) fluoride has also been shown to improve caries in leathery lesions [32]. These clinical studies demonstrate that local application of low concentrations of fluoride is effective for mild to moderate lesions of early root surface caries as well as for noninvasive treatment. 

The pH cycling system used in this study simulates pH changes similar to those in the oral cavity; however, in actual caries, there are individual differences in the buffering capacity of saliva and in the bacterial flora. In addition, the minerals leached from dentin remain in the plaque and accumulate on the surface of dentin; therefore, the behavior of dentin is considered to be different from that of artificial demineralization by pH cycling used in the present study. However, a demineralization-inhibiting effect of fluoride varnish has been reported [31], and the same effect is expected for gel dentin hypersensitivity coating materials. Recently, the remineralization effect of calcium phosphate nanoparticles [33], the caries inhibition effect of silver ions, and the antibacterial effect of zinc chloride [34] have been reported. Therefore, the development of new preventive materials based on ion delivery systems, using hydrophilic gels as the base material, is expected. As pine-resin-based varnish is expected to have an ion-releasing effect, materials that can deliver ions over the medium- to long-term are highly promising agents for reducing the incidence of caries.

## 5. Conclusions

The gel-type hypersensitivity control material used in this study is a new type of agent that shows promise in its ability to control demineralization. Fluorine from this material is deeply absorbed into dentin in a short time; thus, such materials are expected to inhibit dentin demineralization, as with fluoride varnish.

## Figures and Tables

**Table 1:** Fluoride containing the dentin hypersensitivity treatment materials.

Material	Ingredients	Code	Manufacturer
**MS Coat F**	Copolymer with sulfonic acid group	MS	SUN MEDICAL
	Oxalic acid		
	Water		
	Sodium fluoride (3000 ppm)		
**MS Coat** Hys-Block **Gel**	Copolymer with sulfonic acid group	HS	SUN MEDICAL
	Oxalic acid		
	Water		
	Sodium fluoride (900 ppm)		
	Potassium salt		
	Thickener		
**CTX2 Varnish**	Sodium fluoride 5% (22,600 ppm)	FV	Oral Biotech
	Rosin		
	Ethanol		

## Data Availability

Not applicable.
